# Effect of *in vitro* simulated digestion on the anti-*Helicobacter Pylori* activity of different Propolis extracts

**DOI:** 10.1080/14756366.2023.2183810

**Published:** 2023-03-14

**Authors:** Paolo Governa, Giulia Romagnoli, Paola Albanese, Federico Rossi, Fabrizio Manetti, Marco Biagi

**Affiliations:** a Department of Biotechnology, Chemistry and Pharmacy, University of Siena, Siena, Italy; b Department of Physical Sciences, Earth and Environment, University of Siena, Siena, Italy

**Keywords:** Propolis, *Helicobacter pylori*, *in vitro* simulated digestion, urease, molecular docking

## Abstract

*Helicobacter pylori* (HP) is among the most common pathogens causing infection in humans worldwide. Oxidative stress and gastric inflammation are involved in the progression of HP-related gastric diseases, and they can be targeted by integrating conventional antibiotic treatment with polyphenol-enriched natural products. In this work, we characterised three different propolis extracts and evaluated their stability under *in vitro* simulated gastric digestion, compared to their main constituents alone. The extract with the highest stability to digestion (namely, the dark propolis extract, DPE) showed a minimum bactericidal concentration (MBC) lower than 1 mg/mL on HP strains with different virulence factors. Finally, since urease is one of the virulence factors contributing to the establishment of a microenvironment that promotes HP infection, we evaluated the possible inhibition of this enzyme by using molecular docking simulations and *in vitro* colorimetric assay, showing that galangin and pinocembrin may be involved in this activity.

## Graphical abstract

**Figure:**
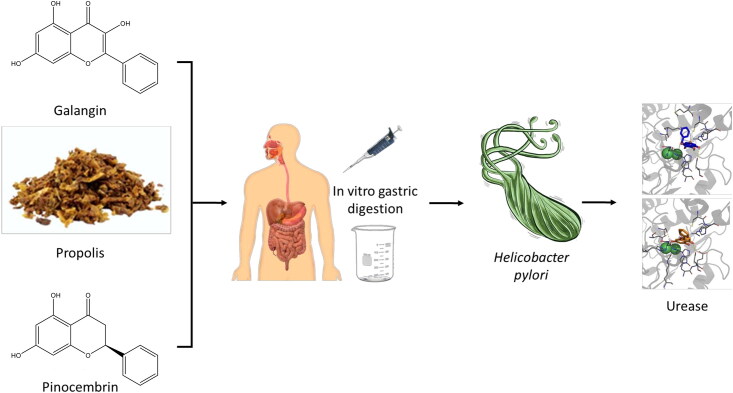

## Introduction

*Helicobacter pylori* (HP), a spiral-shaped Gram-negative microaerophilic bacterium, which colonises the stomach, is among the most common pathogens causing infection in humans worldwide.^1^ The ability of HP to overthrow the host physiology and to evade the immune response complicates the clinical management of the infection.^2^ Indeed, the inability of the immune system to eradicate HP may lead to a progressive clinical course, characterised by the appearance of gastritis, atrophy, intestinal metaplasia, and dysplasia, eventually leading to gastric/duodenal ulcer and gastric cancer.^3^

To colonise the host stomach, HP needs to adapt to the gastric environment, by surviving the acidic pH, penetrating, and growing into the mucus layer.^4^

Different virulence factors, such as the presence of flagella and adhesion proteins, as well as the expression of the vacuolating cytotoxin A (VacA) and cytotoxin-associated gene A (CagA), participate in the HP colonisation phase or are involved in the clinical manifestation of HP infection. Urease is an amidohydrolase enzyme involved in the acid acclimation of the colonisation phase, by catalysing the hydrolysis of urea into ammonia and carbon dioxide, thus increasing the environment’s pH.^5^ Also, it has been shown to participate in the pathogenesis of gastritis induced by HP^6^ and to trigger processes that initiate pro-angiogenic responses, suggesting a role in the development of gastric carcinoma.^7^ Several drug discovery approaches have led to the identification of urease inhibitors,^8^ such as acetohydroxamic acid and flurofamide^9^^,^^10^ However, acetohydroxamic acid has raised safety concerns due to side effects,^9^ while flurofamide has a short half-life at the acidic pH of the stomach.^11^ Thus, the search for novel urease inhibitors is still a demanding task. Interestingly, derivatives of the natural flavonoid diosmin, which were specifically designed to inhibit urease, showed better anti-HP activity, compared to acetohydroxamic acid, used as a reference standard.^12^ Other natural products that have been evaluated for their ability to inhibit urease include Geranium spp., Helleborus spp., and Hyssopus spp. extracts,^13^ isoflavonoids from *Calopogonium mucunoides* Desv.,^14^ indigoferin from *Indigofera heterantha* Brandis,^15^ *Hypericum oblongifolium* Choisy constituents,^16^ and flavanone glucosides from *Galinsoga parviflora* Cav..^17^ Moreover, honey extracts from different sources were found to be efficient inhibitors of Jack Bean urease, which suggest that bee products and their constituents could be used as a source of anti-HP compounds^18^^,^^19^

The current clinical management of HP infection is based on the use of proton pump inhibitors in combination with at least two antibiotic drugs, such as amoxicillin, metronidazole, clarithromycin, tetracycline, and levofloxacin.^20^ Several issues arise with the use of these therapeutic approaches, including the rapid development of bacterial resistance to the antibiotic, particularly to clarithromycin and levofloxacin, the low stability of antibiotics in the acidic environment of the stomach, and the scarce compliance of the patients.^21^ Moreover, oxidative stress and gastric inflammation are strictly involved in the progression of HP-related gastric diseases and are not targeted by conventional antibiotic therapy^22^^,^^23^

To overcome the limitations of conventional antibiotic therapy, natural products with anti-HP activity have been widely investigated.^24^ Some examples include *Aloe vera* L.,^25^ *Allium sativum* L,^26^ sulforaphane isothiocyanates,^27^ *Apium graveolens* L.,^28^ *Glycyrrhiza glabra* L.,^29^ red wine,^30^ resveratrol,^31^ *Origanum majorana* L. and *Citrus reticulata* Blanco,^32^ as well as isolated flavonoids^33^^,^^34^ Another innovative example is represented by carvacrol and thymol which were found to selectively inhibit HP carbonic anhydrases, leading to the impairment of HP biofilm production and the release of outer membrane vesicles.^35^

Propolis is a resinous product made by honeybees to repair and protect the hives, preventing the microbial infection of larvae. Its chemical composition varies depending on the vegetal origin and geographical region where it was produced, affecting the physical properties, such as colour, smell, taste, and consistency. Flavonoids are widely present in most of the propolis, independently from the origin.^36^ Flavones, such as chrysin and acacetin, are mostly present in the temperate region-native propolis, while flavonols, such as galangin and quercetin, are mostly present in Eurasian propolis. Pinocembrin, a flavanone, is commonly found in different propolis, independently from the origin.^36^ Other frequently found constituents include caffeic acid phenethyl ester (CAPE),^37^ phenolic acids (such as benzoic acid, gallic acid, caffeic acid, cinnamic acid, and chlorogenic acid), and terpenes.^36^

Traditionally, propolis has been used for centuries as an antimicrobial agent.^38^ More recently, indeed, this activity has been demonstrated *in vitro*, *in vivo*, and in clinical trials as well, against Gram-positive and Gram-negative bacteria, including drug-resistant species, such as methicillin-resistant *Staphylococcus aureus*, vancomycin-resistant Enterococcus, and several Streptococcus strains, with minimum inhibitory concentration (MIC) varying based on the chemical composition^39^^,^^40^ Antifungal activity was also reported against different Candida species.^41^ The most widely accepted antimicrobial mechanism of action of propolis is related to its effect on membrane permeability, which may lead to cell lysis and may participate in reducing the development of resistance to antibiotic and antifungal drugs.^42^ Also, inhibition of bacterial RNA-polymerase was observed for some of the propolis constituents.^43^

Another important biological effect of propolis consists in its ability to modulate the immune response, leading to a pronounced anti-inflammatory activity,^44^ and its antioxidant properties,^45^ which make it a promising candidate for the management of the complex pathological situation related to HP infection. The anti-inflammatory activity is mainly related to the content in CAPE and galangin. Both were reported to inhibit the nuclear translocation of NF-κB^46^^,^,^47^ which is a key pathway by which CagA promotes gastric inflammation.^48^ On the other hand, the antioxidant and radical scavenging activity of propolis was found to depend mostly on the presence of pinocembrin, chrysin, and pinobanksin.^49^

Information on the *in vitro* anti-HP effect of propolis is emerging^50^ and was also evaluated by our group in the past,^51^ even if the actual clinical effectiveness is still to be validated.

In this work, three different propolis extracts were characterised from a chemical point of view and, for the first time, their stability under *in vitro* simulated gastric digestion was evaluated in comparison to their main constituents alone. The extract that showed the highest stability to digestion was tested for its anti-HP activity. Finally, the possible inhibition of urease was evaluated by using *in vitro* colorimetric assay, molecular docking, and dynamics simulations.

## Material and methods

### Sample preparation and chemical analysis

All solvents were from Sigma-Aldrich (Milan, Italy). Raw green propolis (Bnatural, Corbetta, Milan, Italy) and dark poplar-type propolis (Selerbe, Tavarnelle Val di Pesa, Florence, Italy) were extracted using 80% v/v ethanol for 2 h. A commercial propolis-based extract (Propolfenol®), standardised to contain 50% w/w total phenolic compounds, kindly provided by Erba Vita Group S.p.A. (Chiesanuova, Republic of San Marino) was dissolved in 80% v/v ethanol. Three different propolis extracts were, hence, obtained: green propolis extract (GPE), dark propolis extract (DPE), and Propolfenol^®^ (PPE). The final propolis concentration was adjusted to 400 mg/mL for each extract.

For the chemical characterisation by LC/MSD iQ, samples were further diluted in 80% v/v ethanol to obtain a propolis concentration of 2.5 mg/mL and then filtered. A Single Quadrupole Mass Spec-Agilent instrument, equipped with a Poroshell 120 EC-C18 4.6 × 100 mm, 2,7 µm column was used (LC Column Agilent Technologies, Santa Clara, CA). The mobile phase was composed of water with 0.1% v/v formic acid (A) and methanol with 0.1% v/v formic acid (B), using the following gradient phases: A from 80% at 0 min to 70% at 6 min, from 70% to 50% at 12 min, from 50% to 30% at 15 min, from 30% to 20% at 18 min, from 20% to 10% at 25 min, to 100% of B at 35 min. The column is reconditioned to return it to the initial gradient (80% Solvent A for 6 min) to perform the next run. The flux was set to 0.750 ml/min and the injected volume was 5 µL.

### Absorbance was recorded at 280 nm and 366 nm and calibration curves obtained using galangin and pinocembrin (Sigma-Aldrich) as reference standards (purity > 95%), ranging from 0.008 to 0.5 mg/mL (R2 > 0.99), were used for quantification. In vitro simulated gastric digestion

*In vitro* simulated gastric digestion was carried out as previously described,^52^ with slight modifications. Briefly, extracts (125 µL, corresponding to 50 mg of dry propolis) and reference standards (1 mg/mL) were suspended in 20 ml of simulated gastric juice that contained pepsin from porcine gastric mucosa (300 UI/mL, Sigma-Aldrich) and NaCl (10 mg/mL). The pH of the solution was adjusted to 1.7 using HCl. Samples were incubated for 4 h at 37 °C with shaking. Samples were then filtered and immediately used for further analysis.

### Anti-HP activity

A multiwell suspension test was performed, as previously published,^53^ with a few modifications, for determining the anti-HP activity of the samples. The VacA + CagA + HP clinical isolate 10K, and the VacA + CagA- strain G21 were kindly provided by Professor Natale Figura (Department of Internal Medicine, Endocrine-Metabolic Sciences and Biochemistry, University of Siena, Siena, Italy). Columbia-blood agar (CBA) and Brucella broth bovine serum (BBS) were purchased from Biomerieux (Florence, Italy). Microaerophilic sachets for HP culturing were from Oxoid (Milan, Italy). DPE (13575 − 212 mg/L), galangin (300 − 5 mg/L), and pinocembrin (300 − 5 mg/L) were diluted in BBS and added to the appropriate well of 96-well plates (Sarstedt, Verona, Italy). BBS was used as the negative control. The microbial suspension (1 × 105 cells) was added to each well and plates were incubated at 37 °C for 4 h, in microaerobic conditions. 5 µL of solution were transferred to CBA plates and incubated for 24 h.

The MIC and the minimum bactericidal concentration (MBC) were extrapolated from the multiwell assay and CBA plates, respectively.

The strains susceptibility of the tested HP strains to antimicrobial drugs was confirmed using amoxicillin, clarithromycin, and metronidazole, and comparing MICs with European Committee on Antimicrobial Susceptibility Testing (EUCAST) breakpoint tables (www.eucast.org).

### Urease inhibition assay

The colorimetric urease activity assay kit (Sigma-Aldrich) was used to measure the inhibition of urease activity by DPE, galangin, and pinocembrin. This assay is based on the quantification of urease-produced ammonia, using the Berthelot method.^54^ Briefly, a suspension (1 x 10^6^ CFU/mL) of the 10K clinical isolate was centrifuged (10000 x g for 3 min) and the pellet was used as the source of urease. DPE, galangin, and pinocembrin were used at the MBC found in the previous experiment. 90 µL of DPE (848.4 mg/L), galangin (37.5 mg/L), and pinocembrin (18.8 mg/L), diluted in the assay buffer (10 mM Na3PO4, pH 7.2) containing the 10K suspension pellet (1 x 10^6^ CFU/mL), were added to the appropriate well in a 96-well plate. A standard curve was created by using decreasing NH4Cl concentration (final ammonia concentration 500 − 0 µM). The assay buffer was used as blank control, while the 10K suspension pellet (1 × 106 CFU/mL) in assay buffer was used as the positive control. 10 µL of urea was added to each well and incubated for 15 min at 37 °C. Then, 100 µL of reagent A was added to stop the urease reaction. After mixing, 50 µL of reagent B was added and incubated for 30 min in the dark. Finally, absorbance was measured at 670 nm using a Victor® NivoTM plate reader (PerkinElmer, Waltham, MA). Urease activity was calculated using the following formula:

$$\mathrm{Urease}\;\mathrm{activity}\;(\mathrm{units}/L)=\frac{{\left({\mathrm{Abs}}_{670}\right)}_{\mathrm{sample}}-{\left({\mathrm{Abs}}_{670}\right)}_{\mathrm{blank}}}{\mathrm{Slope}\times t}\times n$$

where Abs_670_ is the absorbance at 670 nm, n is the dilution factor, Slope is the slope obtained by linear regression fitting of the standard curve, and t is the incubation time (15 min).

The percent inhibition of urease was then calculated using the following formula:

$$\mathrm{Urease}\text{ \% }\mathrm{inhibition}=\frac{{\left(\mathrm{Urease}\;\mathrm{activity}\right)}_{\mathrm{positive}}-{\left(\mathrm{Urease}\;\mathrm{activity}\right)}_{\mathrm{sample}}}{{\mathrm{Urease}\;\mathrm{activity}}_{\mathrm{positive}}}\times 100$$

where (Urease activity)_positive_ is the urease activity (units/L) of the positive control.

### Computational details

The X-ray three-dimensional structure of the HP urease with acetohydroxamic acid bound in the active site (PDBID: 1e9y) was obtained from the Protein Data Bank (PDB).^55^

The protein was prepared using AutoDock Tools,^56^ following the standard preparation protocol,^57^ adding polar hydrogens, assigning Gasteiger-Marsili atomic charges^58^ then merging non-polar hydrogens.

The three-dimensional coordinates of galangin and pinocembrin were downloaded from the ZINC database^59^ in Mol2 format and converted to PDBQT format using OpenBabel.^60^

The protein was treated as rigid, and the grid box was centred on the co-crystallized ligand (box centre: x:127.99, y:129.092, z:86.811) and sized to be 14 × 14 × 14 Å.

AutoDock Vina (v1.1.2) was used to perform the molecular docking simulations, setting the exhaustiveness to default.^57^

Molecular dynamics (MD) simulations were conducted using the Desmond software to evaluate the stability of the complex obtained from the docking calculations. The docked complex was placed in a water box, including counterions to neutralise the charge, using a TIP3P solvent model. The system was then minimised to reduce energy, heated to 300 K, and equilibrated to obtain a 100 ns MD trajectory. The simulations were carried out using the default settings for all other parameters.

### Statistical analysis

The statistical differences between the biological results were determined by ANOVA. Values are expressed in the range of ± standard deviation and *p* < 0.05 was considered statistically significant. Graphs and calculations were performed using GraphPad Prism.

## Results

**Figure 1.**
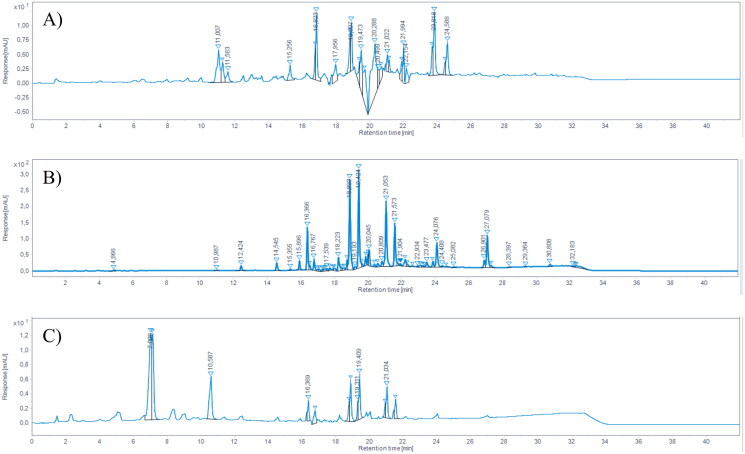
HPLC-DAD chromatograms of (A) GPE, (B) DPE, and (C) PPE, registered at 280 nm. Pinocembrin RT = 18.89; chrysin RT = 19.43, galangin RT = 21.05, kaempferol RT = 21.57.

By using LC/MSD iQ, pinocembrin (RT = 18.89 min) and galangin (RT = 21.05 min) were identified in each sample (Figure 1).

The chemical characterisation of the samples is reported in Table 1. Galangin was found to be the main flavonoid of each propolis sample, while pinocembrin was less abundant in each sample. Dark propolis extract (DPE) contained the highest amount of both galangin and pinocembrin, followed by Propolfenol® extract (PPE) and green propolis extract (GPE), respectively. Other identified compounds, which are typically found in propolis samples, include chrysin (RT = 19.43) and kaempferol (RT = 21.57). For the purpose of this study, only galangin and pinocembrin were quantified, as they are the two typical chemical markers of propolis.

**Table 1.** Chemical characterisation of the extracts. Values (mg/g dry propolis) are expressed as mean ± standard deviation.

Sample	galangin	pinocembrin
GPE	12.65 ± 0.63	1.64 ± 0.15
DPE	83.46 ± 0.59	27.42 ± 0.29
PPE	13.84 ± 0.23	3.11 ± 0.21

When used as a single reference standard, galangin showed high stability to simulated gastric digestion, with a recovery 6-fold higher than pinocembrin (Table 2).

**Table 2.** Relative gastric stability of reference standards. Values (%) are expressed as mean ± standard deviation.

Sample	Gastric stability
galangin	73.97 ± 8.26
pinocembrin	12.31 ± 1.15

**Figure 2.**
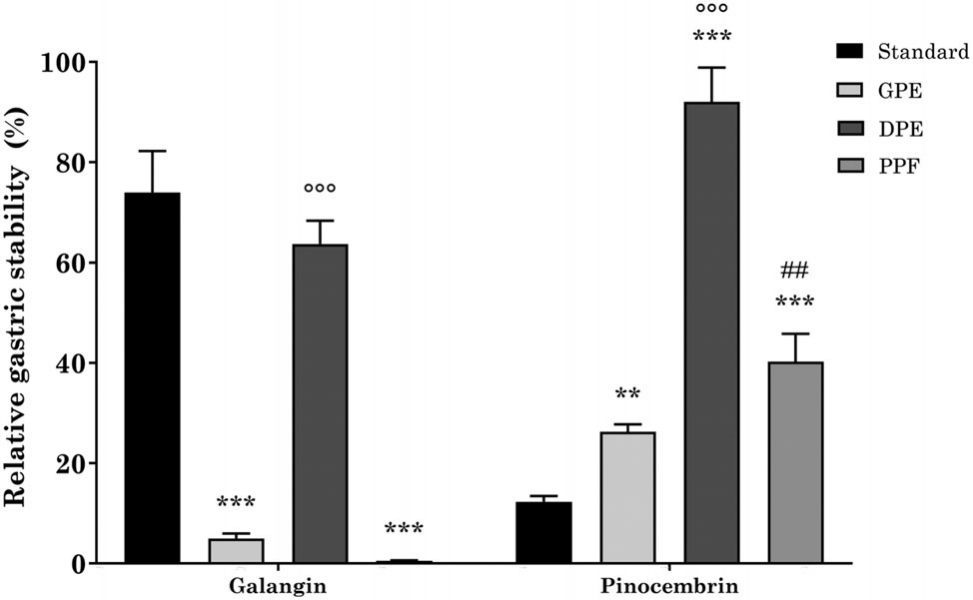
Relative gastric stability of GPE, DPE, and PPE flavonoids. ***p < 0.001 vs reference standard; °°°*p* < 0.001 DPE vs GPE and PPE; ##*p* < 0.01 PPE vs GPE; two-way ANOVA followed by Tukey’s post-hoc.

Different recoveries were obtained when using the three propolis extracts (Figure 2). Indeed, when using GPE and PPE, galangin stability was approximately 5% and 0.5%, respectively, which is significantly lower in comparison to the reference standard alone. Conversely, while being slightly lower than the reference standard, DPE had a protective effect on galangin stability, which resulted to be 63% and significantly higher than GPE and PPE. On the other hand, each propolis extract was able to significantly protect pinocembrin from gastric digestion-induced degradation. Indeed, pinocembrin recovery was 26%, 94%, and 40% when using GPE, DPE, and PPE, respectively. Similarly to galangin, the stability of pinocembrin in DPE was significantly higher than that of GPE and PPE.

Interesting MIC and MBC values, lower than 1000 mg/L, were obtained on both 10K and G21 with DPE. Galangin and pinocembrin, however, gave better results, with MIC and MBC values being 37.5 mg/L and 18.8 mg/L for galangin, and 18.8 mg/L and 37.5 mg/L for pinocembrin, on the 10K clinical isolate and G21 strain, respectively (Table 3). Noteworthy, the MIC and MBC of galangin and pinocembrin were in a similar order of magnitude compared to that obtained with metronidazole.

**Table 3.** Anti-HP activity of DPE and its constituents against the VacA + CagA + 10K clinical isolate and the VacA + CagA- G21 strain. Amoxicillin, clarithromycin, and metronidazole were used as reference drugs. MIC and MBC are expressed as mg/L.

	10K		G21	
Sample	MIC	MBC	MIC	MBC
DPE	848.4	848.4	424.2	424.2
galangin	37.5	37.5	18.8	18.8
pinocembrin	18.8	18.8	37.5	37.5
amoxicillin	0.3	0.3	0.1	0.1
clarithromycin	0.03	0.1	0.01	0.01
metronidazole	4.2	8.4	1.1	2.2

DPE showed a weak anti-urease activity at the tested concentration, with a % inhibition lower than 30%. Conversely, both galangin and pinocembrin resulted particularly active, with more than a two-fold increase of the % inhibition in comparison to DPE (Table 4).

**Table 4.** Urease inhibition by DPE, galangin, and pinocembrin at the respective MBC and IC_50_.

Sample	Concentration (mg/L)	Urease inhibition (%)^a^	IC_50_ (mg/L)^b^	Docking binding energy (kcal/mol)
DPE	848.4	27.8 ± 1.3	1297.2 ± 114.3	
galangin	37.5	68.5 ± 7.1	19.4 ± 1.9	−5.6
pinocembrin	18.8	57.9 ± 4.6	15.7 ± 1.2	−5.6

^a^Helicobacter pylori 10K clinical isolate urease; ^b^Jack bean urease.

These results were confirmed by performing a full dose-response experiment on Jack bean urease. Indeed, the IC_50_ of DPE was about two orders of magnitude higher compared to galangin and pinocembrin, with pinocembrin being slightly more active than galangin (Table 4).

Molecular docking simulations were performed to elucidate the possible binding mode of galangin and pinocembrin in the active site of HP urease.

**Figure 3.**
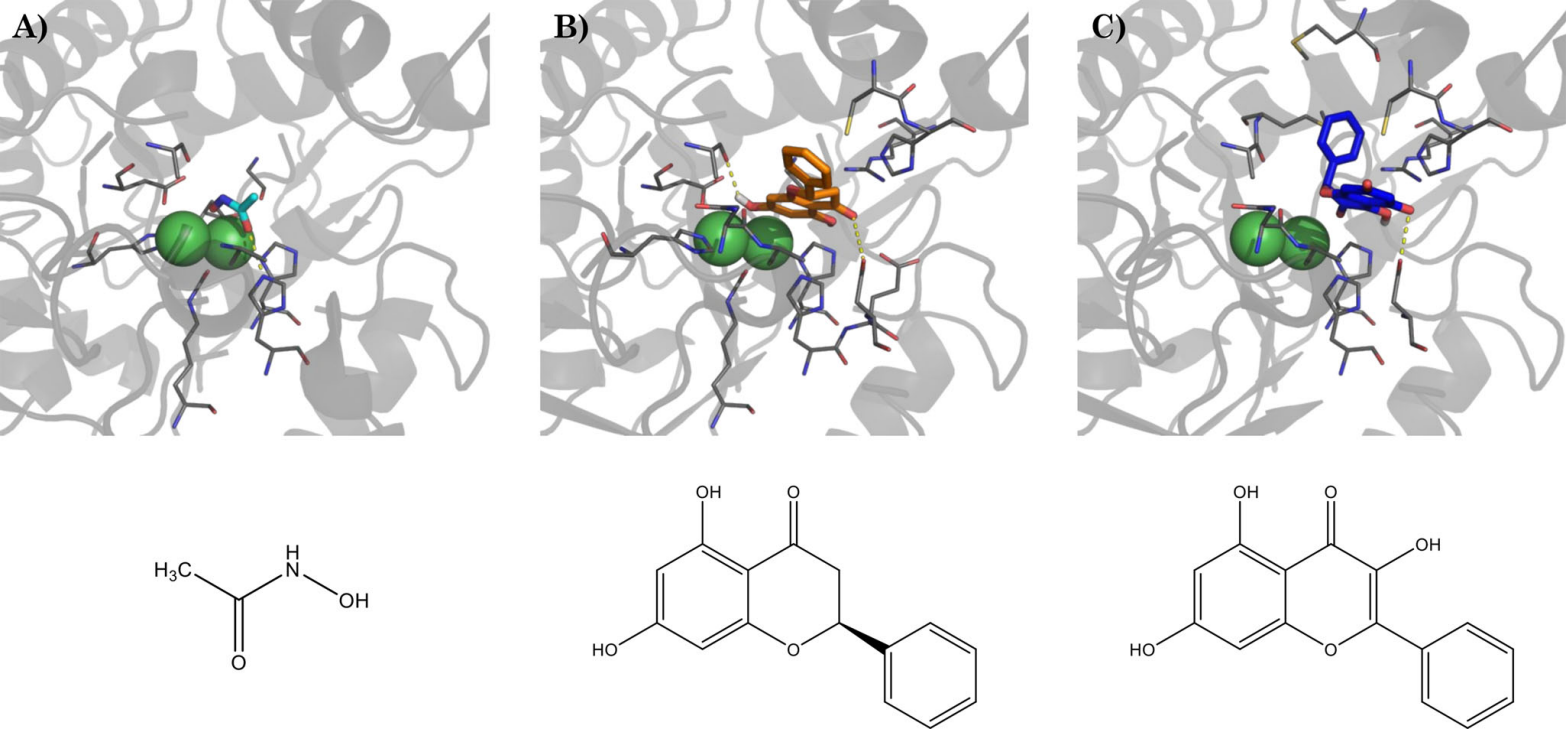
(A) Binding mode of acetohydroxamic acid (cyan) in the active site of HP urease (PDBID: 1e9y), and best docking pose of (B) pinocembrin and (C) galangin. Interacting residues are shown as gray lines. Hydrogen bonds are represented by yellow dashed lines. Bottom: schematic representation of acetohydroxamic acid, pinocembrin, and galangin, respectively.

The co-crystallized ligand, acetohydroxamic acid, binds the active site of urease by coordinating the di-nuclear nickel metallo-centre, which is involved in the decomposition of urea. Also, it makes hydrophobic contacts with Ala169 and Ala365, as well as a hydrogen bond with His221 (Figure 3(A)).

Galangin and pinocembrin, in their top-scored docking pose, result in comparable binding energy (Table 4), but assume different binding poses: pinocembrin binds deep in the active site, close to the two nickel ions, with the B-ring located outside the binding site, and assuming a flat conformation. Two main anchor points are represented by the hydrogen bonds between the 4-OH group and the carboxyl moiety of Asp223, and between the 7-OH group and the backbone carbonyl of Ala365 (Figure 3(B)). Galangin, on the contrary, binds farther from the nickel ions, with the chromone group-oriented in the opposite direction, compared to pinocembrin, and the B-ring perpendicular to it, assuming a binding pose similar to that reported by Xiao and colleagues for quercetin.^61^ Due to the different binding modes, the hydrogen bond with Asp223 occurs through the 5-OH group, while the hydrogen bond with Ala365 is no more possible. Moreover, the B-ring is embedded between Cys321 and Met366 (Figure 3(C)).

To assess the stability of the docked complex and to better evaluate the ligand-protein interactions we performed 100 ns MD simulations. The root-mean square deviation (RMSD) evolution of the 3D coordinates of galangin and pinocembrin atoms during the MD simulation time compared to the last frame of minimisation showed a mean value of 2.55 ± 0.44 Å, and 2.98 ± 0.34 Å, respectively, and an internal ligand fluctuation of 0.43 ± 0.09 Å and 0.26 ± 0.09 Å, respectively, which suggests overall good stability of the complex. By performing cluster analysis of the MD trajectories an additional anchor point, consisting of a hydrogen bond with Glu222, was found for both galangin and pinocembrin, together with an extended network of ionic contacts with His 13, His138, Thr171, Kcx219, His274, and Asp362.

## Discussion

The data obtained in this study suggest that the stability of galangin and pinocembrin in simulated gastric digestion is dependent on their amount in the initial extract. More importantly, even if more data are required to fully understand the reasons why DPE is more effective than GPE and PPE in protecting its main constituents from degradation during gastric digestion, it is evident that dissimilarities in the chemical composition (i.e., the presence and amount of minor constituents, other than galangin and pinocembrin) of propolis extracts from diverse sources may modulate differently the gastric stability of their constituents.

DPE resulted to be the most stable propolis extract after simulated gastric digestion, thus possessing an important characteristic to be exploited for evaluating its anti-HP effectiveness. Although only by two-fold, DPE was found to be more active against the G21 strain, compared to the 10K clinical isolate. Interestingly, MIC and MBC values towards the 10K clinical isolate are identical, and the same was observed towards the G21 strain. This suggested that DPE and its flavonoids may act mainly with a bactericidal mechanism. Consistently, it has been speculated that the antimicrobial activity of propolis is due to its ability to bind the cell wall, leading to the lysis of the microbial cell.^62^ A similar observation was also reported for several flavonoids.^63^

Although DPE gave interesting MIC and MBC values, galangin and pinocembrin gave better results, with galangin being two-fold more active towards the G21 strain, compared to the 10K, and pinocembrin being two-fold more active on the 10K clinical isolate, compared to the G21 strain. Remarkably, the MIC and MBC of galangin and pinocembrin were in a similar order of magnitude compared to that obtained with metronidazole. It is noteworthy that the 10K clinical isolate represents a more relevant model to evaluate the anti-HP activity of test compounds compared to the G21, as it presents both VacA and CagA virulence factors.

By using a turbidimetric method, Skiba and co-authors observed an anti-HP effect of galangin at concentrations between 60 µM and 90 µM,^64^ which is consistent with our results, considering the differences in the method and HP strain used. Moreover, the authors evaluated the cytotoxicity of galangin on human gastric adenocarcinoma (AGS) cells, excluding any possible toxic effect after 16 h incubation. While a similar evaluation on AGS cells is not available for pinocembrin, Zhu and co-workers observed a notably lower cytotoxic effect of pinocembrin on normal immortalised breast epithelial MCF-10A cells compared to three different breast cancer cell lines, thus highlighting its safety of use.^65^ Using an agar diffusion test, Romero and co-authors obtained higher MIC for galangin and pinocembrin (i.e., in the range of 256–512 mg/L and 512–1024 mg/L, respectively, depending on the HP strain used), compared to our work.^66^ This could be explained by the different experimental method, but, most importantly, by the different HP strain used. The 10K clinical isolate used in this work is both VacA + and CagA+, thus representing an ideal model for evaluating the anti-HP activity of a drug candidate. The activity of DPE and its constituents on the 10K clinical isolate suggests a possible role in the prevention of the VacA-induced immune response and may help in reducing the risk of CagA-induced gastric ulcer and cancer.

The gastric localisation of HP is one of the main factors limiting the success of the drug discovery process.^67^ Indeed, to overcome the limited persistence in the stomach due to turnover and emptying time, a compound must be rapidly effective. The bactericidal effect of propolis and its constituents was already evident after 4 h treatment, thus suggesting a quick anti-HP activity. Moreover, as observed in the *in vitro* simulated gastric digestion experiment, DPE was able to protect galangin and pinocembrin from degradation. Hence, DPE could be considered an encouraging candidate for the management of HP infection.

Urease is a virulence factor involved in the colonisation phase of the HP infection.^68^ By using a simple colorimetric method, we determined the *in vitro* inhibition of 10K urease induced by DPE, galangin, and pinocembrin at their respective MIC.

While galangin gave a higher % inhibition, compared to pinocembrin, it should be stressed that samples were tested at their respective MIC. Being galangin MIC twofold higher than that of pinocembrin, urease inhibition by pinocembrin is expected to be higher than that of galangin at the same concentration. Indeed, by using Jack bean urease, we observed that the IC_50_ of pinocembrin was slightly lower in comparison to galangin. Although Jack bean urease has been successfully used as a model for the evaluation of urease inhibitors with anti-HP activity,^69^ a future perspective of this work involves the determination of the IC_50_ of DPE, galangin, and pinocembrin on HP urease, to confirm these results. Two studies by Baltas and co-workers investigated the inhibition of HP and Jack Bean urease by several propolis extracts of different origin, reporting a wide IC_50_ range between 80 and 1560 mg/L^50^^,^,^70^ which are in line with the concentration of DPE used in this work. In 2012, Xiao and colleagues evaluated the effect of 20 flavonoids on HP urease activity, observing IC_50_ values between 11.2 µM and 4628 µM.^61^ Through structure-activity relationship analysis, they also highlighted 3-OH, 5-OH, and 3′,4′-dihydroxyl groups as important for urease inhibitory activity. We calculated the IC_50_ values of galangin and pinocembrin, which both possess the 5-OH group, on Jack bean urease and they fitted very well in this range. The different binding orientation assumed by galangin and pinocembrin in their top-scored docking pose and after 100 ns MD simulations may explain the slight differences in the urease inhibitory activity.

## Conclusion

The current pharmacological management of HP infection is not ideal, as bacterial resistance phenomena are arising. Moreover, the side effects of the anti-HP therapy, such as nausea, vomiting, and gastrointestinal disorders, limit the patient’s compliance. Thus, there is a need for the development of novel treatments, to be administered alone or in combination with other anti-HP drugs, which should be able to overcome this condition.

In this work, we tested the ability of chemically characterised propolis extracts to act as anti-HP agents. We found that DPE may represent an interesting candidate, as it contains a high amount of galangin and pinocembrin, which seem to be involved in the anti-HP activity of propolis. Interestingly, both these flavonoids possess a rapid bactericidal activity, which is pivotal for counteracting the limited persistence in the stomach. Moreover, an additional therapeutic advantage is conferred by the inhibition of HP urease, a virulence factor involved in the colonisation phase of the HP infection. The higher stability in the gastric environment may suggest that galanin may be a better anti-HP candidate, compared to pinocembrin. Nevertheless, when using DPE, the gastric stability of pinocembrin significantly increases. Hence, the administration of an active dose of DPE is expected to maintain both galangin and pinocembrin levels above their effective anti-HP concentration.

In conclusion, we identified galangin and pinocembrin as interesting drug candidates for the management of HP infection. Antibiotic treatment is still essential for the eradication of HP; thus, we suggest the possibility to administer these compounds in combination with conventional antibiotic therapy, to reduce the risk of bacterial resistance development. Finally, as the stability to digestion of natural products is highly influenced by the co-administration of other constituents, such as in the case of herbal extracts, galangin, and, particularly, pinocembrin should not be administered as isolated compounds. The use of a chemically characterised propolis extract may be favourable, as it helps maintaining the active concentration of these flavonoids for the whole gastric digestive process.

## Supplementary Material

Supplemental Material
