# Supplementary material for: Effect of in vitro simulated digestion on the anti-Helicobacter Pylori activity of different Propolis extracts
Source: J Enzyme Inhib Med Chem. 2023 Mar 14;38(1):2183810. doi: 10.1080/14756366.2023.2183810 (PMC10026752; doi:10.1080/14756366.2023.2183810)
Supplement: Supplemental Material [file IENZ_A_2183810_SM8068.pdf]

## Supplemental information

# Effect of *In Vitro* Simulated Digestion on the Anti-*Helicobacter Pylori* Activity of Different Propolis Extracts

Paolo Governa[a]*, Giulia Romagnoli[a], Paola Albanese[b], Federico Rossi[b], Fabrizio Manetti[a], Marco Biagi[b]

[a]*Department of Biotechnology, Chemistry and Pharmacy – Department of Excellence 2018-2022, University of Siena, Siena, Italy;* [b]*Department of Physical Sciences, Earth and Environment, University of Siena, Siena, Italy*

*Paolo Governa, Department of Biotechnology, Chemistry and Pharmacy, University of Siena, Via A. Moro, 2, I-53100, Siena, Italy; email: paolo.governa@unisi.it; Phone: +39 0577 234307; ORCID: 0000-0002-5976-780X; Twitter: @paologoverna;

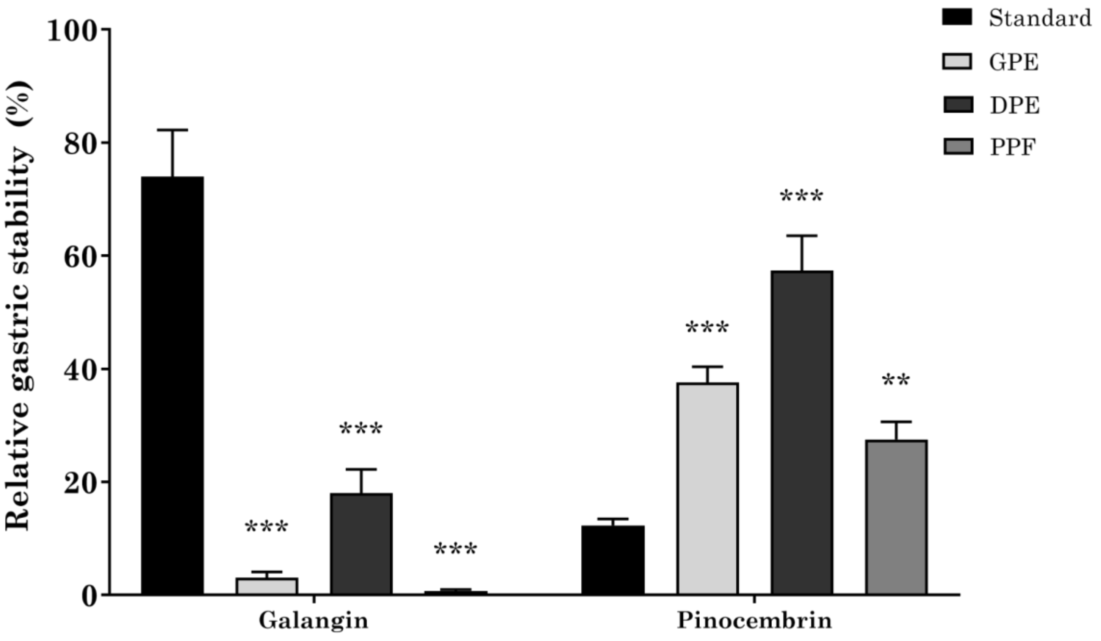


Figure S1. Relative gastric stability of GPE, DPE, and PPE flavonoids, when used at the same concentration of the reference standards (1 mg/mL). ***p<0.001 vs reference standard; two-way ANOVA followed by Tukey’s post-hoc.

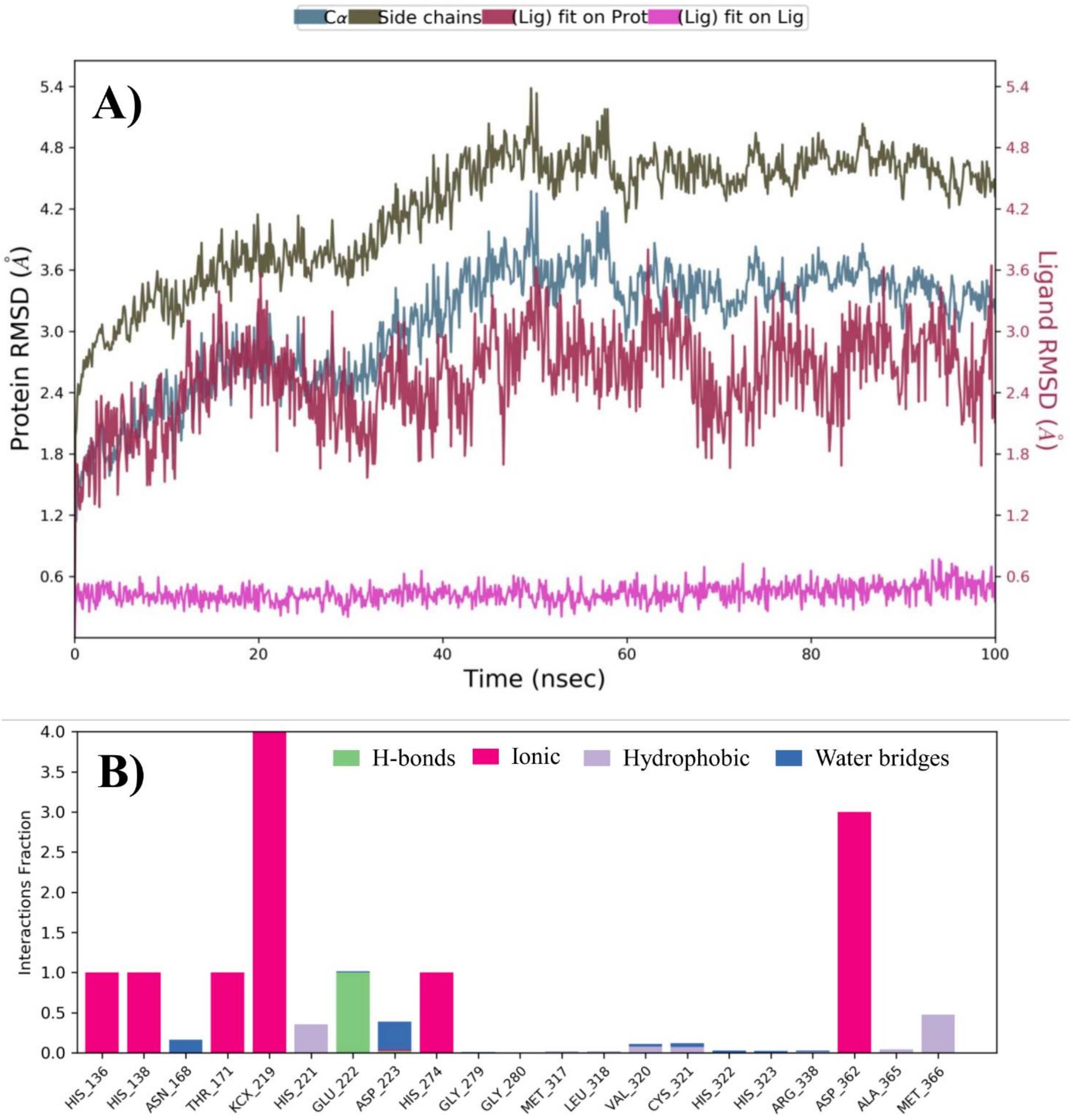


Figure S2. (A) Protein-ligand RMSD and (B) protein-ligand contacts of the urease-galangin complex during 100 ns MD simulation.

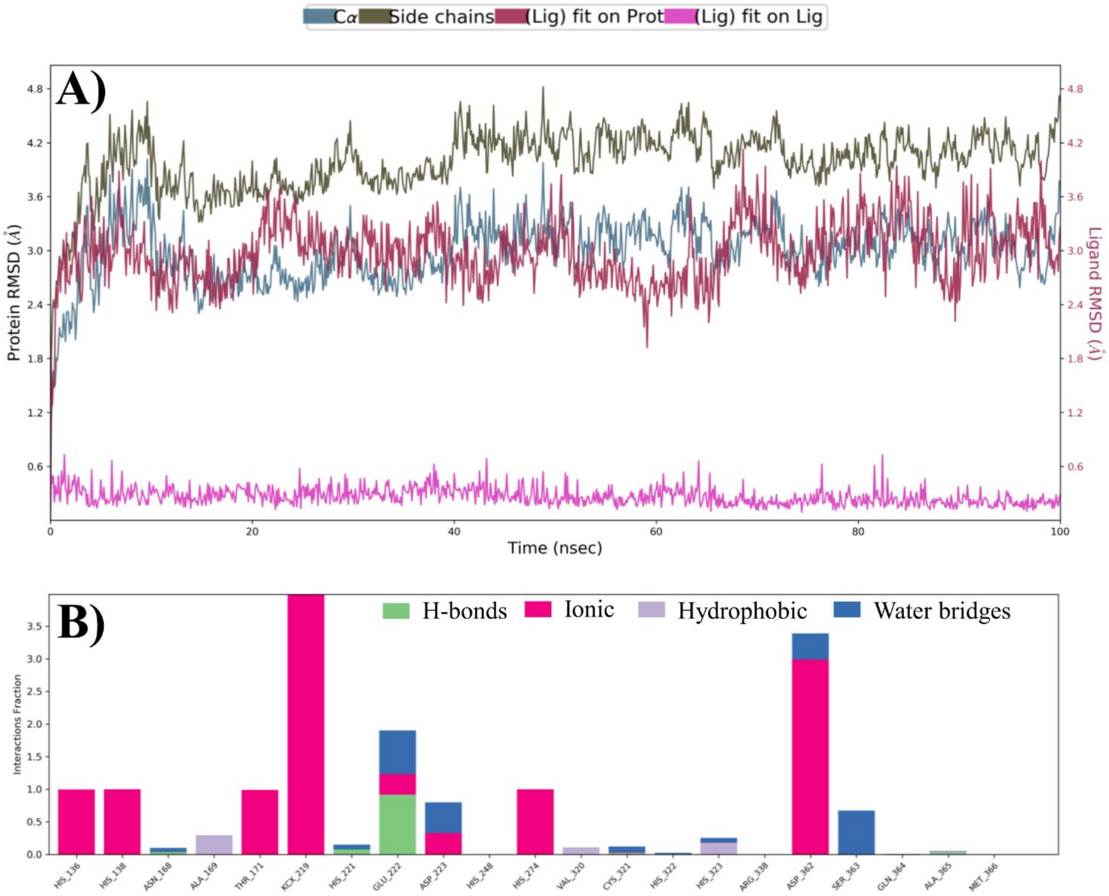


Figure S3. (A) Protein-ligand RMSD and (B) protein-ligand contacts of the urease-pinocembrin complex during 100 ns MD simulation.

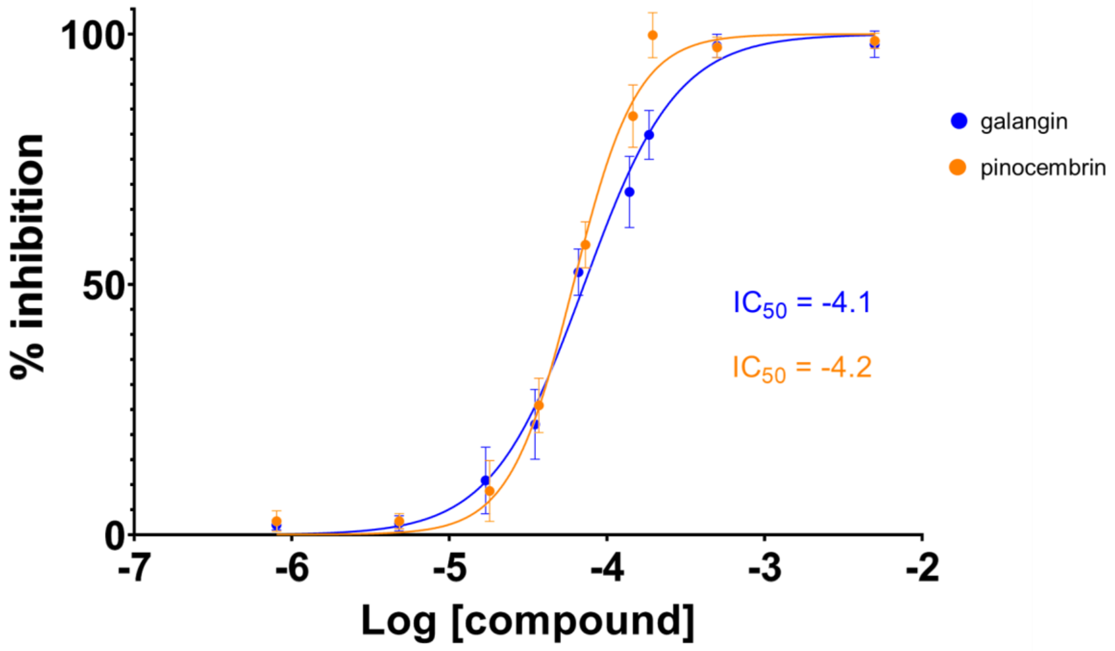


Figure S4. Dose-response curve of galangin and pinocembrin and their $IC_{50}$.
